# Clinical Characteristics of Hepatitis B Virus-Associated Hepatocellular Carcinoma Patients in Southwest Nigeria

**DOI:** 10.3390/pathogens14020169

**Published:** 2025-02-08

**Authors:** Vivian N. Nwude, Olufunmilayo A. Lesi, Charles Onyekwere, Emilie Charpentier, Judith M. Hübschen

**Affiliations:** 1 Department of Internal Medicine, Carver College of Medicine, University of Iowa, 200 Hawkins Drive, Iowa City, IA 52242, USA; 2 Department of Internal Medicine, College of Medicine, University of Lagos, Lagos 12003, Lagos State, Nigeria; 3 Lagos State University Teaching Hospital, 1-5 Oba Akinjobi Way, Ikeja 101233, Lagos State, Nigeria; charles.onyekwere@lasucom.edu.ng; 4 Clinical and Applied Virology, Department of Infection and Immunity, Luxembourg Institute of Health, 4354 Esch-sur-Alzette, Luxembourg; emilie.charpentier@lih.lu

**Keywords:** hepatitis B virus, hepatocellular carcinoma, Osteopontin, Nigeria

## Abstract

Hepatitis B virus (HBV)-associated hepatocellular carcinoma (HCC) is a major cause of morbidity and mortality in West Africa, but its presentation is poorly understood. In this study, we describe the clinical characteristics of HBV-associated HCC patients in Lagos, Nigeria. Data for all cases were collected at the emergency and gastroenterology units (2017–2019), considering chronic carriers as controls. Clinical data and routine biochemical and radiologic test results were extracted from the files. The serum biomarkers (Osteopontin, AFP-L3, DCP) were investigated. For some cases, the hepatitis B viral load was determined. The mean age of the cases (*n* = 92) was 41.4 years, compared to 39.9 years for the controls (*n* = 100). Clinically, 69.5% of cases presented with ascites, 66.3% had nodules occupying >50% of the liver, and 67.4% had moderate hepatic encephalopathy. The mean viral load and the median values of Osteopontin, AFP-L3, and DCP for the cases were significantly higher than for the controls (*p* < 0.001). The area under the curve, sensitivity, and specificity were significantly higher for Osteopontin, compared with DCP and AFP-L3 (*p* < 0.001). Most HCC patients presented at a late disease stage, when the prognosis is usually poor. Especially Osteopontin seems to have potential for early HCC detection and could possibly complement AFP and abdominal ultrasound scan for risk-group screening.

## 1. Introduction

The hepatitis B virus (HBV) infection is a global public health problem, with an estimated 2 billion people having been infected sometime during their lives, and approximately 257 million suffering from a chronic hepatitis B (CHB) infection [1,2]. Most cases of a HBV infection occur in developing countries, with Sub-Saharan Africa having the second largest burden, after Southeast Asia [3]. The prevalence of CHB infection in Nigeria is 9.5%, making it highly endemic, with an estimated 20.03 million people currently being affected [4]. In developed countries, 90% of HBV infections are cleared spontaneously as they are mostly acquired in adulthood, whereas in the developing countries, HBV is transmitted in the perinatal and childhood period, with 90% of infected new-borns developing a CHB infection. Without treatment, 30% progress to hepatocellular carcinoma (HCC) [5]. HCC is the fifth most frequently diagnosed malignancy, and the second leading cause of cancer-related deaths in men worldwide [6]. HCC contributes most to primary liver cancer, accounting for 75% to 85% of cases. Liver cancer was the third and sixth leading cause of mortality in men and women, respectively, in 2022 [7]. The number of deaths from HCC per year is virtually identical, underscoring the high fatality rate. CHB is the most important risk factor for HCC, contributing to more than 50% of HCC cases worldwide, and 70–80% of HCC cases in HBV high-endemic regions [5]. Early HCC is often asymptomatic and is devoid of pathognomonic features, making the diagnosis very challenging without efficient surveillance systems. The progression of untreated HCC and the associated clinical features have been well characterised in the developed countries, where the patients with HCC present at very early stages of the disease and are usually in the 6th to 7th decade of life [8]. This contrasts with the pattern observed in the developing countries, where almost all patients with HCC present at earlier ages and at very late stages of the disease. Yet, limited data exist about the clinical presentation of HCC patients in many African countries, including Nigeria [9,10,11]. Late diagnosis results in very poor prognosis and limits the treatment options [8] and highlights the facts that at-risk individuals are not identified, comprehensive HCC surveillance programmes are lacking, and the access to expert medical care is limited. Additionally, the absence of trust and financial protection in health-care systems in Africa leads to a lack of health-seeking behaviour [8,10].

According to the international guidelines, an abdominal ultrasonography, combined with serum alpha-fetoprotein (AFP) every 6 months, is recommended as HCC surveillance regimen in patients at risk. The sensitivity of the ultrasound combined with AFP for diagnosing early-stage HCC was reported to be only 63% (95% CI, 48–75%) [12]. Hence, the role of other biomarkers (e.g., osteopontin, *Lens culinaris* agglutinin AFP–L3 and Des-y-carboxy prothrombin (DCP)) in the detection of early HCC has been evaluated, and their sensitivities and specificities were found to be higher than AFP when used alone or in combination with ultrasonography. However, little to nothing is known about the usefulness of these biomarkers in Sub-Saharan Africa.

Hepatitis B viral factors, including HBV genotypes and viral load, have been documented as strongly predictive of the clinical outcome [13]. HBV load is increasingly recognized as a prognostic factor in the presence of established HCC [14], and has even been proposed as the strongest independent predictor of death after cirrhosis [15]. Viral load is not routinely investigated in patients with HBV-associated HCC in Nigeria and other parts of Africa, due to the high cost and the lack of available kits in the region. As such, little is known about the correlation between the viral load and the clinical outcome in that setting. HBV is differentiated into ten recognized genotypes (A–J). In Africa, genotype D is supposed to dominate in the North, E in the West and A in the East [16,17]. In Nigeria, with its high HBV prevalence, very few recent genotyping studies are available. Genotyping studies from 2008 found genotype E to be predominant [16]. Recent studies conducted in different regions of the country showed mixed infections, with multiple patterns of genotypes, including genotype E (most predominant) and genotypes B, C and D (least predominant) [18].

This study investigated the clinical, laboratory, and virological characteristics of patients with HBV-associated HCC in Nigeria, compared to matched asymptomatic chronic carriers.

## 2. Materials and Methods

### 2.1. Ethical Considerations

The study was approved by the Ethical Review Board of the Lagos University Teaching Hospital (NHREC: 19/12/2008a).

### 2.2. Selection of Study Subjects

Patients between 18 and 75 years, who presented at the emergency and gastroenterology outpatient clinic of a tertiary health centre in Lagos, Nigeria, between 2017 and 2019, diagnosed with HBV-associated HCC and without a history of antiviral treatment, were recruited as cases after informed written consent. All cases had a chronic HBV infection (HBsAg positive for longer than 6 months), were anti-HBc IgG positive, anti-HBc IgM negative, and anti-HBs negative, without co-morbidities (such as hepatitis C virus (HCV) and human immunodeficiency virus (HIV) infections). A HCC diagnosis was confirmed histologically and/or via ultrasound-detected hepatic mass lesion, showing a characteristic arterial hypervascularity, combined with a venous washout on a contrast computerized tomography (CT) scan, a magnetic resonance imaging (MRI), or a combination of alpha-fetoprotein (AFP) levels greater than 400 ng/mL with an ultrasound hepatic mass lesion.

Patients recruited as controls after informed consent were, as much as possible, age- and sex-matched CHB carriers, who presented at the gastroenterology outpatient unit of the same institution, with no history of prior antiviral therapy and no co-morbidities. They fit the criteria for a CHB inactive carrier state, as defined by AASLD (American association for the study of liver disease) [19], namely HBsAg positive for >6 months, HBeAg negative and anti-HBeAg positive, serum HBV viral load < 2000 IU per mL, normal ALT level and liver biopsy or imaging confirming absence of chronic liver disease. In addition, they had AFP levels < 400 ng/mL.

A structured questionnaire was used to record the patients’ socio-demographic information, familial, environmental, medical and social risk factors, as well as relevant clinical findings.

### 2.3. Sample Collection

Ten millilitres of whole blood were obtained from each study participant via aseptic venepuncture into two 5 mL sterile EDTA vacutainer tubes, immediately after recruitment. Samples were transported upright in a cold box to the laboratory within 2 h of collection and were centrifuged at 3000 rpm, for 10 min, to obtain plasma. Plasma from each participant was aliquoted into three 2 mL sterile cryovials (Sarstedt AG & Co. KG, Nümbrecht, Germany) and stored at −80 °C until testing. All specimens were labelled with unique laboratory codes for confidentiality.

### 2.4. Laboratory Tests

Routine serological and chemistry test results were obtained from the laboratory reports and the case files of all participants. Serological tests for HBsAg, HBeAg, antiHBe, antiHBc and antiHBs (Aria Diagnostics, Indianapolis, IN, USA), AFP (Roche Cobas E411, Roche, Mannheim, Germany), HIV 1/2 ((Alere Determine, Abbott Laboratories, Chicago, IL, USA) and Stat Pak (Chembio Diagnostic Systems Inc., New York, NY, USA)), HCV (Monalisa anti-HCV kit v 3.0, Bio-Rad Laboratories, Inc., Marnes-la-Coquette, France), Bilirubin, Albumin, alanine transaminase (ALT), aspartate amino transferase (AST) and alkaline phosphatase (Roche Cobas C311 platform, Roche, Germany) were performed. The detection of total HDV-antibodies was conducted as part of this study using a commercial assay (DRG Diagnostics GmbH, Marburg, Germany) according to the manufacturer’s instructions.

Osteopontin was measured with the Osteopontin Human ELISA kit (BioVendor, Brno, Czech Republic), AFP-L3 with the AFP-L3 ELISA Kit 96 (Cusabio, Wuhan, China) and DCP with the Human Abnormal prothrombin (APT)/Des-γ-carboxy prothrombin (DCP) ELISA Kit (MyBioSource, San Diego, CA, USA).

HBV DNA was extracted using the COBAS AmpliPrep Instrument (Roche Diagnostics, Mannheim, Germany) for automated specimen processing. The COBAS TaqMan 48 Analyzer (Roche Diagnostics, Germany) was then used for automated amplification. HBV detection and quantification were achieved with the COBAS AmpliPrep/COBAS TaqMan HBV Test kit, version 2.0 (Roche Diagnostics, Germany). Fresh DNA was extracted for genotyping using the QIAamp DNA mini kit (Qiagen, Hilden, Germany). Two semi-nested PCR reactions were used to amplify the pre–S and S fragments, as described before [17,20]. Sequencing and sequence analysis were performed as previously mentioned [17], but using MEGA version 7 [20]. The newly generated sequences were submitted to DDBJ/EMBL/GenBank under accession numbers OQ082342–OQ082555.

Data were analysed using the IBM statistical package for social science (SPSS) software version 26. Categorical variables were presented with frequencies and percentages, while the numeric variables were represented as means. Kolmogorov Smirnov test was used to assess data normality assumptions.

Associations between the categorical variables were assessed using Chi-square and Fisher exact tests. Mean comparison between the two groups was conducted using an independent Student’s *t*-test, while the Mann–Whitney test was used to compare median values. Receiver operating characteristic (ROC) was used to generate the ideal cut off for biomarkers. Area under curve (AUC), sensitivity, and specificity were also calculated. The *p*-value for significance was set at <0.05 at 95% confidence interval.

## 3. Results

### 3.1. Demographic, Biochemical, Haematological, and Serological Characteristics of Participants

The ages of the subjects ranged between 21 and 70 years, with a mean of 41.4 ± 10.5 years in the cases and 39.9 ± 9.9 years in the controls (*p* = 0.442, Table 1). The highest proportion of cases (59.7%) and controls (68%) belonged to the 31–40 years and 41–50 years age groups. The lowest proportion of cases (5.4%) and controls (2%) was between 61- and 70-years-old. The male to female ratio was approximately 2:1 and 1.4:1 among the cases and controls, respectively (*p* > 0.173). More than half of the cases (60.9%) were married while about three quarters of the controls (74%) were single (*p* = 0.052).

Yoruba ethnicity was the most common among the cases (55.4%) and controls (59%), while Hausas were the least common in both groups (9.8% and 7%, respectively). The ethnicity among the cases and controls was not significantly different (*p* = 0.619).

As expected, based on the selection criteria, liver biochemistry, lipid profile, AFP and white cell counts were different between the controls and cases, with the latter having deranged values generally.

The platelet count was on average lower among cases (200.89 ± 59.7) than controls (223.2 ± 47.3, *p* = 0.37), while the international normalized ratio (INR) was slightly higher (1.22 ± 0.3 versus 1.09 ± 0.2, *p* = 0.07, Table 1).

Both the controls, for which HBeAg negativity was a selection criterion, as well as the cases, were HBeAg negative.

Only four participants (one control and three cases, *p* = 0.813) had antibodies to the hepatitis D virus.

### 3.2. Clinical Characteristics of Cases

A total of 63 cases had ascites; 31.5% had mild ascites, while approximately 26% presented with moderate ascites. About 66.3% of the patients had nodules involving less than 50% of the liver on the abdominal CT scan (Table 2).

The majority (88%) of the patients were in Child–Pugh class C, and none were in class A. Using the Okuda staging system, most of the patients were in stages 2 (42.4%) and 3 (43.5%) of the disease, while only 14% were in stage 1. Using the West Haven classification, the majority (67.4%) of the patients had moderate hepatic encephalopathy (Table 2).

### 3.3. Viral Load in Cases

The mean viral load for cases was 208,930 ± 21 copies/mL (range 200 to 67,608,297), and the median was 171,530 copies/mL.

A total of 81 (88%) subjects had viral loads > 10,000 copies/mL, with the highest frequency recorded within the age groups 31–40 years and 41–50 years (25 cases each; *p* = 0.863). The number of male cases with viral loads > 10,000 copies/mL was more than twice as high as that of females (57 versus 24, *p* = 0.289, Table 3).

### 3.4. Hepatitis B Viral Load and Clinical Characteristics

The cases in Okuda stages 2 and 3 had higher median viral loads of 548,192 and 171,080 copies/mL, respectively, than cases in stage 1, though not statistically significant (*p* = 0.415, F = 1.76, Table 4).

The median viral load of cases in class C of the Child–Pugh Score was a little higher than cases in class B (176,080 versus 171,530 copies/mL, *p* = 0.823, F = −0.224, Table 4).

### 3.5. Genotyping Results

**Figure 1:**
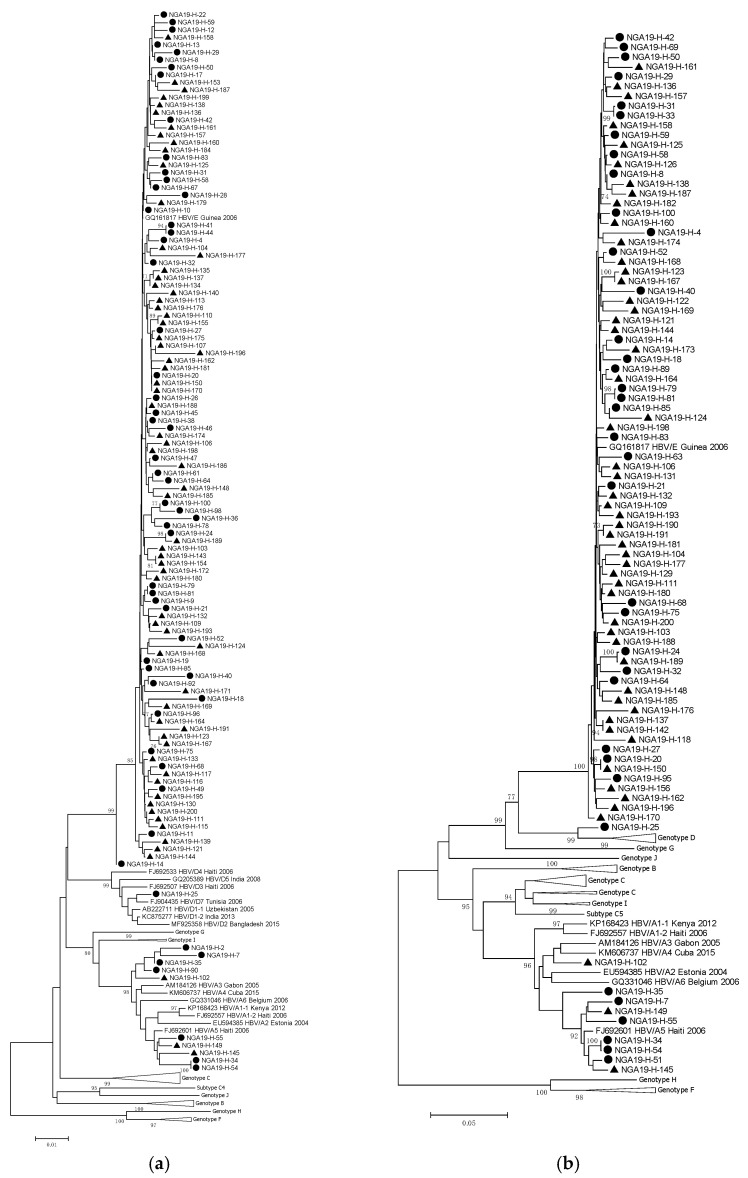
Phylogenetic tree based on a region spanning (**a**) 783 nucleotides of the S fragment and (**b**) 901 nucleotides of the pre-S fragment. The analyses are based on the Neighbor-joining and Kimura 2-parameter methods, and bootstrap values of at least 70 (1000 re-samplings) are shown. Cases are marked with black dots and controls are marked with black triangles.

The S PCR yielded fragments for sequencing from 124 participants (57 cases and 67 controls), while the Pre-S PCR yielded fragments from 90 participants (37 cases and 53 controls). One HCC patient, a 42-year-old female from the western part of Nigeria (NGA19-H-25), was infected by a genotype D strain, and eight cases (NGA19-H-2, 7, 34, 35, 51, 54, 55 and 90) and three controls (NGA-H-102, 145 and 149) were infected by genotype A strains. Genotype E was detected in the remaining cases and controls (Figure 1).

#### 3.5.1. Median Concentration of Biomarkers in Cases and Controls

The median values of Osteopontin, ALP-L3 and DCP concentration were significantly higher in cases than in controls (*p* < 0.001, Table 5).

#### 3.5.2. Receiver Operating Characteristics for Biomarkers of HCC

**Figure 2:**
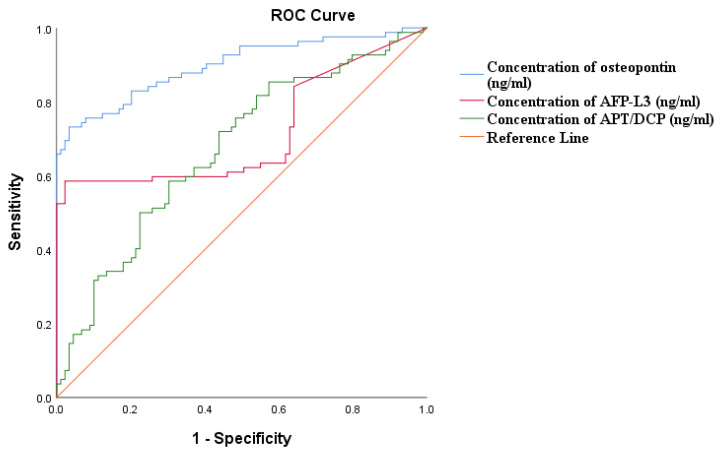
Receiver operating characteristics (ROC) curve for three biomarkers of HCC.

The receiver operating characteristics (ROC) curve shows the diagnostic ability of the three biomarkers, at different concentration threshold points. Osteopontin had the largest area under the curve (AUC = 0.897) and has the highest overall diagnostic accuracy for detection of HCC, with sensitivity and specificity values of 0.829 and 0.796, respectively. However, all biomarkers showed significant utility in diagnosing HCC (*p* < 0.001, Figure 2 and Table 6).

## 4. Discussion

In this study, a mean age of about 41 years was recorded among the cases, with approximately twice as many males as females, similar to previous reports from South-Western Nigeria [21,22], while an even lower mean age of about 33 years at presentation was reported from other Sub-Saharan countries [23]. In contrast, in resource-rich countries, HCC patients are generally older (about 50–60 years), with metabolic causes (alcohol or obesity), and an HCV infection acquired in adulthood is mainly responsible [3]. Our results suggest that HBV-related HCC contributes to the low life expectancy in Nigeria, with a negative impact on the workforce, and thus economic productivity.

The biochemical and haematological parameters were largely deranged in almost all the cases, which is similar to findings in other parts of the world [24,25], and might be explained by the late stage and the severity of the disease at presentation.

Serum alpha-fetoprotein (AFP) is widely used as a diagnostic marker for HCC in West Africa. However, controversies exist about appropriate AFP levels needed to increase the sensitivity and specificity of a HCC diagnosis [26,27]. Our study used a cut-off of 400 ng/L which gave a sensitivity of 89.1%. Although AFP levels of more than 400 ng/mL have been shown to strongly support the presence of HCC, some patients still have normal AFP levels, suggesting that normal or moderately elevated levels cannot exclude HCC diagnosis [9,28].

While studies in Mongolia, Sweden, and the United States, as well as meta-analyses, have found an association between HDV positivity and HCC incidence [29,30,31,32], we and others [33,34] observed a low HDV prevalence in Nigeria, suggesting that a co-infection does not play a major role in the progression to HCC in this setting.

In our study, we used the Okuda and Child–Pugh staging systems since they include common and simple clinical parameters that can easily be measured. Although the Cancer of the liver Italian Program (CLIP) system was recommended for primary staging of liver disease by international and national authorities [9], we were unable to use it because we lacked some of the required parameters. In addition, many patients included in our study presented at late disease stages and were often too sick for further investigations. Okuda staging and Child–Pugh scoring systems showed that two thirds of cases presented at intermediate to advanced stages of disease. This is similar to what was found 14 years earlier in the same institution, when 68% of the HBV-associated HCC patients presented in late stages according to Child–Pugh scoring [35] and suggests little or no improvements concerning disease awareness and early access to hepatitis B testing and treatment since then. Similar to our findings, reviews conducted in two tertiary centres in Saudi Arabia also revealed that the majority of patients (80%) presented in Class B and C according to the Child–Pugh scoring system [9]. Receipts of referrals from all over the country, and the weak implementation of guidelines for surveillance of patients at risk of developing HCC were reasons attributed to the delay of the diagnosis and have probably also played a role in our study. In Nigeria, the high costs of the treatment might be an additional reason patients present late, or sometimes not at all, to the hospital.

Many studies have established that an early diagnosis of HCC (Child–Pugh Class A and Okuda Stage 1) improved survival. However, the stage of liver disease correlated better with survival than the tumour stage [36], suggesting that liver disease stage should be the main factor when making a decision regarding the therapy and counselling of patients.

The most prevalent HBV genotype in this study, like in previous studies in Nigeria, was genotype E [16,17]. Genotype A was found in about 5% of cases and 0.02% of controls and genotype D in a single case only. The clinical significance of genotypes has mostly been studied in settings where genotypes other than genotype E were predominant, and suggested that the risk of progression to chronicity is higher in patients infected with genotypes A and D, than with genotype E [37,38].

The mean load of the HBV DNA for the cases was 208,930 copies/mL, similar to the findings of Jin-Yong Zhou et al. [14], who investigated a comparable patient cohort in South Korea. Our study observed a trend of higher viral loads in the later stages of the disease and in individuals between 31 and 40 years of age. A study on Turkish natives revealed very high viral loads in even younger age groups, of up to 30 years, but no information was provided about the presence of HBeAg, which is associated with an actively replicating HBV infection [39]. In our study, 11% of cases had viral load values below 10,000 copies/mL, above which treatment is started for the chronic HBV infection carriers with deranged AST and ALT levels in Nigeria. Studies have recorded a two- to seven-fold higher risk of HCC at HBV DNA levels of about 10,000 copies/mL, and recommended starting antiviral treatment at lower values, or even irrespectively of the viral load [40,41]. This underscores the need to take other sensitive parameters assessing the liver status into consideration when deciding who should be treated.

In this study, the diagnostic ability, sensitivity, and specificity to detect HCC of the three biomarkers (Osteopontin, ALP-L3 and DCP) were evaluated. Although the median concentrations of all three biomarkers were significantly higher in cases than in controls (*p* < 0.001), Osteopontin had the highest area under the curve (AUC = 0.897), suggesting that it has a high potential as a diagnostic marker in the Nigerian setting. Other studies observed similar AUC, specificity, and sensitivity. These studies also compared the sensitivity of these new biomarkers with AFP alone and when combined, and found that they presented better sensitivity and specificity when paired with AFP [42,43]. We could not compare the sensitivity and specificity of these biomarkers with AFP, because AFP was part of the diagnostic criteria when choosing cases. However, since all these biomarkers have potential of detecting early HCC, further studies in Sub-Saharan Africa are required to assess whether their sensitivity and specificity can even be increased when combined with AFP and an abdominal ultrasound scan.

In conclusion, this study showed that most patients with HCC presented at a late disease stage, when the prognosis is usually poor. This suggests that more awareness campaigns and screening opportunities are required for the timely identification of chronic hepatitis B carriers. Osteopontin in particular seems to have a good potential for detecting HCC and could possibly complement AFP and abdominal ultrasound scans when screening at-risk groups. More studies, especially prospective ones, are needed to evaluate the use of the new markers for early detection of HCC in Sub-Saharan Africa.

## Figures and Tables

**Table 1:** Socio-demographic and laboratory parameters of participants.

	Cases *n* = 92 (%)	Controls *n* = 100 (%)	*p*-Value
**Age (Years) Mean**	41.4 ± 10.5	39.9 ± 9.9	0.442
**Sex**			
Male	63 (68.5)	59 (59.0)	0.173
Female	29 (31.5)	41 (41.0)	
**Marital status**			
Married	56 (60.9)	74 (74.0)	0.052
Single	36 (39.1)	26 (26.0)	
**Ethnicity**			
Hausa	9 (9.8)	7 (7.0)	0.619
Igbo	17 (18.5)	17 (17.0)	
Yoruba	51 (55.4)	59 (59.0)	
Others	15 (16.3)	17 (17.0)	
**Liver Biochemistry**			
ALT (IU/L) 0–45	53.75 ± 4.1	9.45 ± 2.7	<0.001 *
AST (IU/L) 0–35	51.26 ± 6.4	9.36 ± 2.1	<0.001 *
ALP (IU/L) 30–120	161.36 ± 23.0	85.81 ± 21.3	<0.001 *
GGT (IU/L) 0–30	75.38 ± 17.6	22.10 ± 7.0	<0.001 *
Albumin (g/L) 40–60	22.85 ± 3.3	43.88 ± 5.1	<0.001 *
Total bilirubin (mg/dL) 0.3–1.2	4.19 ± 1.9	1.18 ± 0.7	<0.001 *
**Haematology**			
Platelet Count (×10^9^/L)/150–400	200.89 ± 59.7	223.20 ± 47.3	0.373
INR 0.8–1.2	1.22 ± 0.3	1.09 ± 0.2	0.071
AFP (ng/L) 40	2735 (940–7299)	3.09 (1.4–4.0)	<0.001 *
White cell count	10.14 ± 4.8	6.13 ± 1.2	<0.001 *
**LipidProfile**			
Total cholesterol	228.96 ± 14.1	192.82 ± 3.5	<0.001 *
Triglycerides	96.03 ± 14.4	69.28 ± 6.0	<0.001 *
HDL cholesterol	44.41 ± 2.7	72.00 ± 10.8	<0.001 *
LDL cholesterol	150.75 ± 21.8	106.17 ± 9.3	<0.001 *

ALT: Alanine aminotransferase; AST: Aspartate aminotransferase; ALP: Alkaline phosphatase; GGT: Gamma-Glutamyl Transferase; INR: International normalized ratio; AFP: Alpha-feto protein; HDL: High density lipids; LDL: Low density lipids.* statistically significant value.

**Table 2:** Clinical characteristics of cases.

Clinical Characteristics	Cases (*n* = 92)	(%)
**Ascites** None	29	31.5
Mild	29	31.5
Moderate	24	26.1
Gross	10	10.9
**Proportion of liver covered with nodules on imaging**		
≤50	61	66.3
>50	31	33.7
**Child–Pugh Scoring System** **A**	0	0
B	11	12.0
C	81	88.0
**Okuda staging**		
1	13	14.1
2	39	42.4
3	30	43.5
**Hepatic encephalopathy**		
Mild	15	16.3
Moderate	62	67.4
Severe	15	16.3
Coma	0	0

**Table 3:** Association between viral load and selected socio-demographic characteristics among cases.

	≥10,000 Copies/mL (*n* = 81)	<10,000 Copies/mL (*n* = 11)	*p*-Value
**Age groups (Years)**			
21–30	11 (84.6)	2 (15.4)	0.863
31–40	25 (92.6)	2 (7.4)	
41–50	25 (89.3)	3 (10.7)	
51–60	16 (84.2)	3 (15.8)	
61–70	4 (80.0)	1 (20.0)	
**Sex**			0.289
Male	57 (90.5)	6 (9.5)	
Female	24 (82.8)	5 (17.2)	

**Table 4:** Median Viral Load and Clinical Characteristics.

	Median Viral Load (Q1–Q3)	F-Value #	*p*-Value
**Okuda Staging**			
1	95,254 (60,123–1,359,506)	1.757 ^+^	0.415
2	548,192 (100,000–1,495,186)		
3	171,080 (25,060–552,000)		
**Child–Pugh score**		−0.224 *	0.823
B	171,530 (171,080–548,192)		
C	176,080 (25,060–1,394,506)		

^+^ Kruskal Wallis test used * Mann–Whitney U test used; Q1 = First quartile; Q3 = Third quartile. # F-value is significant when >3.89.

**Table 5:** Median Concentration of Biomarkers in Cases and Controls.

	Cases (*n* = 82) Median (Q1–Q3)	Controls (*n* = 89) Median (Q1–Q3)	*p*-Value
**Osteopontin (ng/mL)**	80.00 (37.5–80.0)	15.82 (10.3–27.1)	**<0.001**
**ALP-L3 (ng/mL)**	20.00 (0.5–20.0)	1.83 (0.0–3.0)	**<0.001**
**DCP (ng/mL)**	1.65 (1.4–1.9)	1.42 (1.3–1.6)	**<0.001**

ALP-L3 = *Lens culinaris* agglutinin—reactive fraction of AFP; DCP = Des-y carboxyprothrombin; Q1 = First quartile; Q3 = Third quartile.

**Table 6:** Area under the curve (AUC), sensitivity and specificity for biomarkers of HCC.

	AUC (95% CI)	Sensitivity	Specificity	*p*-Value
Osteopontin	0.897 (0.849–0.946)	0.829	0.796	**<0.001**
AFP-L3	0.716 (0.633–0.798)	0.707	0.728	**<0.001**
DCP	0.671 (0.590–0.752)	0.732	0.631	**<0.001**

## Data Availability

The original contributions presented in this study are included in the article. Further inquiries can be directed to the corresponding author.
